# *Chlamydia suis* undergoes interclade recombination promoting Tet-island exchange

**DOI:** 10.1186/s12864-024-10606-6

**Published:** 2024-07-26

**Authors:** Helena Seth-Smith, Sankhya Bommana, Deborah Dean, Timothy D. Read, Hanna Marti

**Affiliations:** 1 Institute of Veterinary Pathology, University of Zurich, Zurich, Switzerland; 2 Division of Infectious Diseases and Global Health, Departments of Medicine and Pediatrics, University of California San Francisco School of Medicine, San Francisco, CA USA; 3 Joint Graduate Program in Bioengineering, University of California Berkeley, University of California San Francisco, Berkeley and San Francisco, CA USA; 4 Division of Infectious Diseases, Department of Medicine, Emory University School of Medicine, Atlanta, GA USA; 5 Department of Human Genetics, Emory University School of Medicine, Atlanta, GA USA

**Keywords:** *Chlamydia*, *Chlamydiaceae*, Antibiotic resistance, Horizontal gene transfer, Homologous recombination, Tetracycline, Comparative phylogenetics

## Abstract

**Background:**

The obligate intracellular bacterial family *Chlamydiaceae* comprises a number of different species that cause disease in various vertebrate hosts including humans. *Chlamydia suis*, primarily found in the gastrointestinal tract of pigs, is the only species of the *Chlamydiaceae* family to have naturally gained tetracycline resistance (TetR), through a genomic island (Tet-island), integrated into the middle of chromosomal *invasin*-like gene *inv*. Previous studies have hypothesised that the uptake of the Tet-island from a host outside the *Chlamydiaceae* family was a unique event, followed by spread among *C. suis* through homologous recombination. In vitro recombination studies have shown that Tet-island exchange between *C. suis* strains is possible. Our aim in this study was to gain a deeper understanding of the interclade recombination of the Tet-island, among currently circulating *C. suis* field strains compared to in vitro-generated recombinants, using published whole genome sequences of *C. suis* field strains (*n* = 35) and in vitro-generated recombinants (*n* = 63).

**Results:**

We found that the phylogeny of *inv* better reflected the phylogeny of the Tet-island than that of the whole genome, supporting recombination rather than site-specific insertion as the means of transfer. There were considerable differences between the distribution of recombinations within in vitro-generated strains compared to that within the field strains. These differences are likely because in vitro-generated recombinants were selected for a tetracycline and rifamycin/rifampicin resistant background, leading to the largest peak of recombination across the Tet-island. Finally, we found that interclade recombinations across the Tet-island were more variable in length downstream of the Tet-island than upstream.

**Conclusions:**

Our study supports the hypothesis that the occurrence of TetR strains in both clades of *C. suis* came about through interclade recombination after a single ancestral horizontal gene transfer event.

**Supplementary Information:**

The online version contains supplementary material available at 10.1186/s12864-024-10606-6.

## Background

*Chlamydia* (*C*.) *suis* belongs to the *Chlamydiaceae* family, Gram-negative, obligate intracellular bacteria that are primarily responsible for respiratory, ocular and urogenital disease in humans and animals [1]. *C. suis* is a pig pathogen and has mostly been associated with mild clinical signs such as conjunctivitis, diarrhoea but also reproductive disorders to a limited degree [2]. Like many of the *Chlamydiaceae*, *C. suis* is a zoonotic pathogen and has been detected in the eyes, pharynges and rectum of people that come in close contact with pigs, namely farmers and slaughterhouse workers [3–5], as well as in the eyes of trachoma-endemic populations that domesticate pigs [6].

While the clinical impact of *C. suis* on porcine health is mild, *C. suis* has received attention in recent years as the only *Chlamydiaceae* species to have naturally obtained an antibiotic resistance gene: the tetracycline resistance-conferring *tet*A(C) gene [7, 8]. This gene has been detected in many different *C. suis* strains from Europe, Israel and the USA [1]. Where present, it is consistently identified to be part of an over 12 kilobase pair (kb) genomic island termed the Tet-island, likely derived from a plasmid originating from Proteobacteria, located within the chromosomal invasin-like gene *inv* [9, 10]. The nearly 4 kb *inv* gene has an unknown function, and is disrupted by the Tet-island insertion [8, 11]. The complete *inv* gene is only found in two chlamydial species, *C. suis* and *C. caviae*, a pathogen found in guinea pigs. In *C. muridarum* and human *C. trachomatis*, the two closest phylogenetic relatives of *C. suis*, the *inv* gene is truncated or entirely absent, respectively [11, 12].

Discrepancies between the mutation rate of the Tet-islands and the chromosomes led to the hypothesis that *tetA*(C) acquisition in *C. suis* happened relatively recently, and certainly after the separation of the species into two major ancestral clades [9, 10]. Given current data on sequenced strains, the Tet-island possibly originated in the USA before being later transferred to European *C. suis* strains [9, 10]. Moreover, based on the unique structure of the Tet-island and its invariable position within the *C. suis* chromosome, it has been hypothesised that the original acquisition was a rare and possibly singular horizontal gene transfer (HGT) event followed by spread of the Tet-island among *C. suis* strains through homologous recombination [9, 10]. This hypothesis presupposes the occurrence of interclade recombination events after the Tet-island was integrated into *C. suis*. Intra- and interspecies recombination is well-recorded for many *Chlamydiaceae* species [13], however the extent of interclade recombination has so far not been analysed in detail for *C. suis*.

In this study, we investigated the extent of interclade recombination, in both currently available sequences from field isolates, and in vitro-generated recombinants. We performed phylogenetic comparisons of the *inv* gene to that of the Tet-island and of the whole genome using all available *C. suis* genomes, paying special attention to interclade recombination events. Second, we used an established co-culture model [14] using tetracycline-sensitive (TetS) and tetracycline-resistant (TetR) *C. suis* strains from the two major clades to investigate interclade recombination and Tet-island transfer dynamics in vitro.

## Materials and methods

### Generation of in vitro-generated recombinants

The detailed protocol has already been published [14]. Briefly, tetracycline-sensitive, rifamycin-resistant strains S45 RIF, 94 Ry and 111 Ry were individually co-cultured with SWA-141 (4-29b), SWA-107 (5-27b) or SWA-110 (1-28b) in LLC-MK2 cells (continuous Rhesus monkey kidney cell line, kindly provided by IZSLER Brescia, Italy) at 37 °C and 5% CO_2_. After the first passage, selection was added using inhibitory concentrations of rifampicin (Merck, Darmstadt, Germany) or rifamycin (Merck), and tetracycline (Merck). After the second passage, plaque assays were performed as previously described to obtain individual inclusions [15]. Putative recombinants were initially identified by strain-specific PCR followed and confirmed by stability assays for five to ten passages in the presence and absence of selective antibiotics and PCR re-analysis of passaged cultures. Whole-genome sequencing was performed on a subset of these confirmed recombinants, and the recipient strains, using the Illumina MiSeq platform [14].

### Sequence analysis

All read data for field strains [10] and recombinant strains [14] were obtained from SRA or were deposited under PRJNA668469 (Table S1). The complete assembled genome of strain 8-29b (NZ_FTQU01000001) was used as the reference against which to map all reads within CLC Genomics Workbench v20.0.2 and also generate a single nucleotide polymorphism (SNP) phylogeny with parameters that differed from the default as: variant calling with 10x minimum coverage, 10 minimum count and 70% minimum frequency (after which mean coverage was assessed), and SNP tree creation with 10x minimum coverage, 10% minimum coverage, 0 prune distance and including multi-nucleotide variants (MNVs). Genome consensi were extracted from mapped reads using a minimum coverage of 5, a multiple sequence alignment (MSA) was created from the whole genome alignments, exported and run through Gubbins v3.0.00 with 5 iterations. Results were viewed in Artemis [16] and Phandango [17]. FastBaps v 1.0.8 [18] was used to calculate the population structure. BactDating v1.1.1 was run on the gubbins output with up to 5 million iterations and the arc model (Table S6). Traces did not converge, and between three runs, most likely root dates varied from − 7433.75 [-20318.78;-109.86] to -5626.65 [-13128.00;24.31], with mu ranging from 2.57e + 00 [7.44e-01;7.72e + 00] to 2.93e + 00 [1.09e + 00;8.30e + 00].

The *inv* gene was reconstructed from previous assemblies [10] and the Tet-island, where present, was excluded from the resulting MSA, which was generated using MUSCLE [19] in AliView v 1.27 [20]. A phylogeny was created using RAxML (https://raxml-ng.vital-it.ch/) using defaults and 100 bootstraps.

The Tet-island phylogeny was generated from the previous alignment, corrected for the strains used in the whole genome phylogeny (*n* = 22), using RAxML as above.

Phylogenies were compared using phylo.io [21].

### Analysis of in vitro-generated recombinants

Gubbins alignment of in vitro-generated recombinants and all recipient/donor strains was performed. The alignment was then used for subsequent analysis with the Geneious Prime software (v. 2023.1.1; Biomatters, Auckland, New Zealand). Specifically, we extracted individual recombinants and their associated recipient strains and identified recombinations using the “Find Variation/SNPs” function. All areas with three or more SNPs were identified and marked as motifs. Recombinant regions were then curated following comparison of recipient, donor and recombinant using MAFFT alignment followed by the “Find Variation/SNPs” function and curation of identified motifs. Areas with high variation between all three strains were excluded from downstream recombinant analysis. Genes at the predicted ends of the recombinations were identified following alignment against recombinant strain 217.1, which was annotated using Prokka [22].

### Statistical analysis

We used GraphPad Prism (v. 10.0.2, GraphPad Software, Boston, MA, USA) for statistical analyses. Statistical tests included either one‑way ANOVA (Kruskal‑Wallis test) with Dunn’s multiple comparisons test for multiple means, or the Mann‑Whitney test if two groups were compared.

## Results and discussion

### Whole genome phylogeny of field isolates

**Fig. 1:**
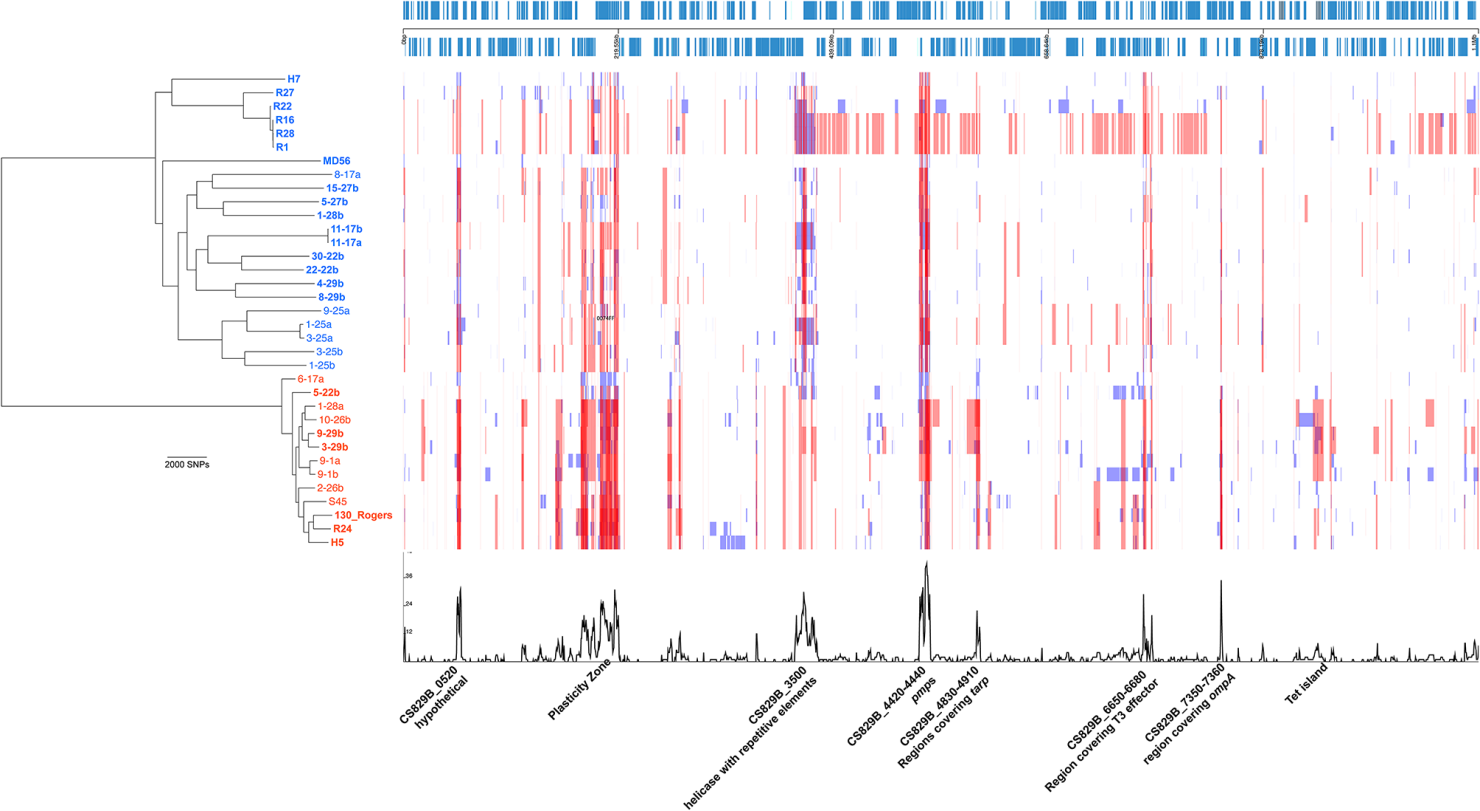
Phylogeny of *C. suis* field strains (*n* = 35) and identified recombinations. The phylogenies with recombinations removed (left) are coloured with Clade 1 in blue and Clade 2 in orange (FastBAPS). Names in bold define strains that carry the Tet-island. The sample names are matched to the genome length tracks (right) where red bars indicate recombinations identified in more than one genome, and blue bars show those only identified in single genomes. The genome with annotated coding sequences (CDSs in blue, both forward and reverse reading frames) of strain 8-29b is shown above these tracks. Below the tracks is a plot of recombination density within the phylogenies, with peaks annotated according to the relevant genome regions, CDSs and predicted encoded proteins. Data around the Tet-island illustrates that not all strains carry the island; *inv* fragments at both sides are commonly predicted to be involved in recombinations. The data was generated using Gubbins [23] and visualised using Phandango [17]

Available genome data from tetracycline-resistant and -sensitive isolates (“field strains”) were collected (*n* = 41, Table S1), from previous publications [9, 10]. All genomes with under 15x mean read coverage were excluded (*n* = 6) providing *n* = 35 genomes to analyse. The phylogeny (Fig. 1) closely agrees with that already published [10], despite the use of alternative analysis tools. Bayesian clustering was used to confirm the separation into two clades. Recombination analysis of the collection of field strains also identified highly impacted loci (Fig. 1), including the Tet-island, as previously described. Bayesian dating of the ancestor of the species was attempted, giving results unfortunately with high uncertainty (see methods). The most recent common ancestor appears to have emerged several thousand years before the common era; further data is required to improve upon this analysis.

### Comparisons of phylogenies of whole genomes, insertion site *inv* and Tet-island of field strains

**Fig. 2:**
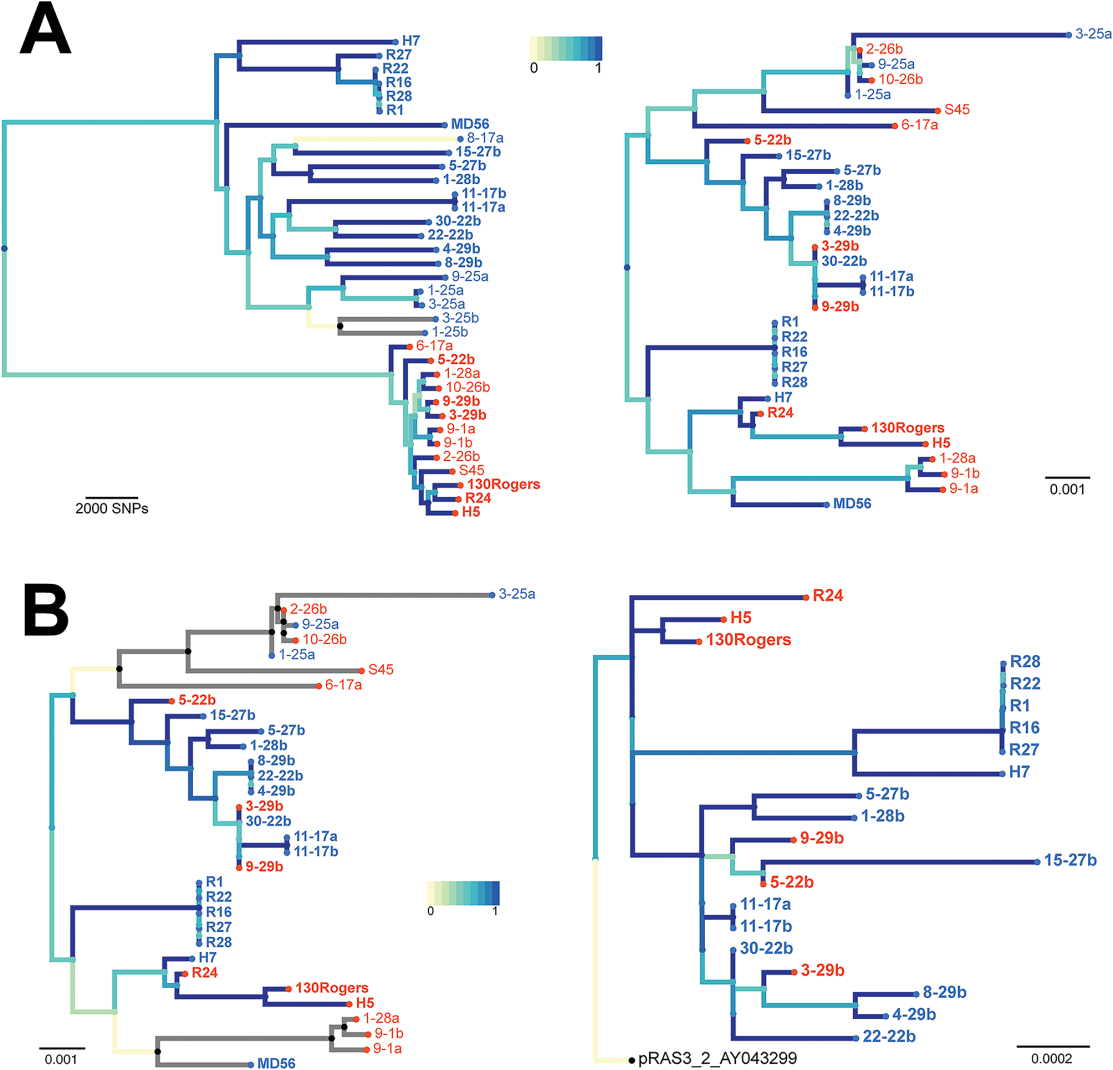
Comparison of phylogenies. The colour scale of the branches shows the weighting of the similarities. Names in bold define strains that carry the Tet-island. **A**: Whole genome (left) vs. *inv* (right) phylogenies. **B.*** inv* phylogeny (left) vs. Tet-island phylogeny (right; [10]). Leaves are coloured with Clade 1 in blue and Clade 2 in orange

The complete *inv* gene was reconstructed from genomes containing disrupted versions of the gene, and a phylogeny of *inv* was created to investigate the degree of agreement with a whole genome phylogeny with recombination events removed (Fig. 2A). A high degree of clade mixing is clear in the *inv* phylogeny, which does not reflect the whole genome phylogeny, and suggests recombination of *inv*. A comparison of the phylogeny of *inv* with that of the Tet-islands from Seth-Smith et al. [10], shows a much higher congruence (Fig. 2B), strongly suggesting that the *inv* gene is linked to the Tet-island during Tet-island movement, and not that it serves purely as an integration site. This is particularly clear between clades and speaks for recombination as a means of Tet-island transfer.

### Analysis of recombination sites in field and in vitro recombinant isolates

**Fig. 3:**
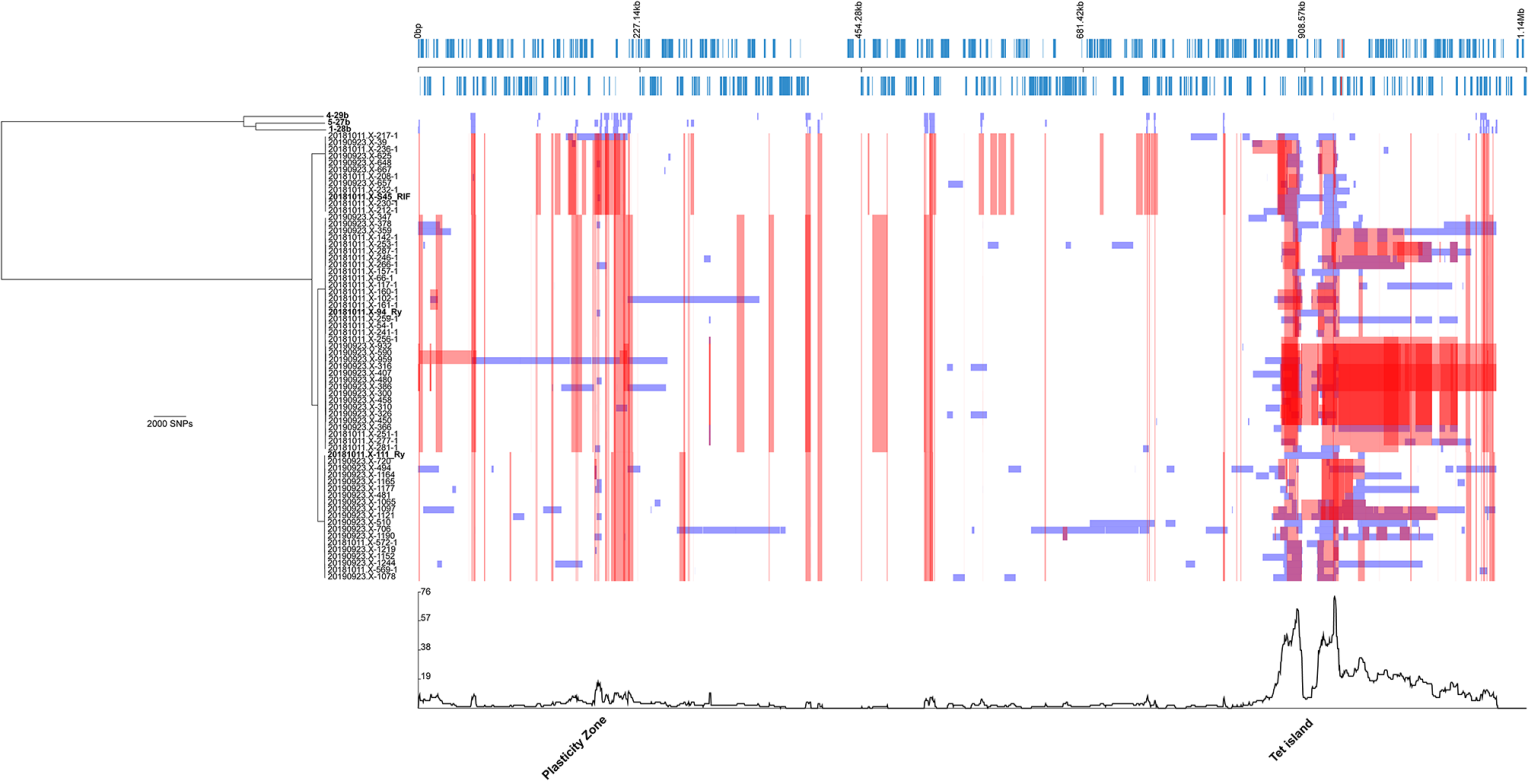
Phylogeny of *C. suis* in vitro-generated recombinant strains *n* = 63 including Tet-carrying (*n* = 3) and derived resistant parental (*n* = 3) strains (bold) and identified recombinations. The phylogenies with recombinations removed (left) have bold names representing parental strains. The sample names are matched to the genome length tracks (right) where red bars indicate recombinations identified in more than one genome, and blue bars show those only identified in single genomes. Where recombinations are identified in more than one strain (red recombination blocks), this is either due to recombinations identified between the parent strains (Figure S1), or to duplicate picking of plaques from the same parent combination. The genome with annotated coding sequences (CDSs in blue, both forward and reverse reading frames) of strain 8-29b is shown above these tracks. Below the tracks is a plot of recombination density within the phylogenies, with peaks annotated according to the relevant genome regions, CDSs and predicted encoded proteins. The data was generated using Gubbins [23] and visualised using Phandango [17]

A phylogeny of in vitro recombinant genomes from previous plaque-purified strains generated from Tet-island transfer experiments [14] (*n* = 63, all over 25x mean read coverage), including the six parental strains was generated, to compare against that of the field strains (Fig. 3). The recombinant strains cluster phylogenetically around the recipient parental strains, all of which are from Clade 2 (SWA-94 is 10-26b; SWA-111 is 1-28a; and S45), compared to the Clade 1 Tet-donor strains (SWA-141 is 4-29b; SWA-107 is 5-27b; and SWA-110 is 1-28b) (Fig. 1). The distribution of recombinations within the in vitro-derived recombinants is clearly different from that within the field strains. The in vitro-derived recombinants were selected on the basis of having acquired the Tet-island in a rifamycin/rifampicin resistant background. Hence the largest peak of recombination is across the Tet-island with the downstream recombination site extending further relative to the Tet-island compared to the upstream recombination site. The predicted mean recombination size across all recombinations is 10.6 kb, ranging between 4 bp and 135 kb (Table S2), which stands in contrast to comparison of the field strains where the average recombination size was predicted to be much shorter with 1.1 kb (2 bp-21.2 kb, Table S3).

We could not identify consistent upstream or downstream recombination junctions involving the Tet-island of the in vitro-derived recombinants, suggesting that a general homologous, rather than site-specific, recombination mechanism is responsible. Further recombinations across the genome are also apparent, despite not having been selected for, showing the promiscuity of recombination under permissive conditions.

### Recombinations in in vitro-generated recombinants reveal high variability in selected and non-selected areas

Predicted recombinations within in vitro-generated strains were analysed in detail by finding the variations between individual recombinants with their respective parental strains using Geneious Prime (Table S4). Overall, we identified a total of 237 recombinations with a mean size of 26.6 kb (range: 20 bp to 492 kb). We detected an average of 3.8 recombinations per in vitro-generated strain (*n* = 63), all of which included one recombination involving the Tet-island; recombinations across this region were notably longer than regions that were not under specific selective pressure, with a mean size of 9.5 kb (20 bp to 193 kb) and 73.8 kb (15.2–492 kb) for recombinations without and with the Tet-island, respectively. This confirms the results of our previous study which contained a smaller sample size [14]. These findings are also in line with a co-culture study co-infecting *C. muridarum* and a tetracycline-resistant *C. suis* which identified a 98 kb recombinant region involving the Tet-island [24]. In co-infection experiments that did not include the Tet-island and corresponding tetracycline selection, average recombinant regions were variable. One study concerning *C. muridarum / C. trachomatis*, produced recombinant region sizes similar to those in our study, ranging between 558 bp and 124 kb [25], while other studies investigating *C. trachomatis* yielded longer regions ranging between 200 and 400 kb [26, 27].

We then investigated recombinations involving the Tet-island relative to their distance to *inv* (Table S5). There was no significant difference in the recombination extension length upstream and downstream (*p* = 0.7). However, while the upstream recombination site is an average of 17.1 kb upstream of the proximal *inv* fragment (56/63) and never further upstream than 53 kb, the downstream recombination site ranges between 0 kb and 440 kb from the distal fragment of *inv*. The reason for this finding is unclear. In *Chlamydia*, both homologous recombination pathways—double-strand break-dependent RecBCD and single-strand break-dependent RecFOR—have been identified [13]. One unknown component is the identity of Chi sites, the site where the double-stranded degradation of RecBCD ends and single-strand degradation begins for the binding of RecA [13]. It is possible that there are one or more Chi sites close to the upstream *rrn* operon resulting in more constrained recombination sites compared to recombination downstream of the Tet-island. However, we do not have sufficient data to support this hypothesis.

### Limitations

Sample and whole genome sequencing of further Tet-island carrying and tetracycline-sensitive isolates of *C. suis* would better inform all aspects of this study.

In the current study, all in vitro recombinations were performed with a Tet donor in Clade 1 and a recipient in Clade 2. To further investigate interclade recombination, Tet donors and recipients in reverse clades would prove interesting.

## Conclusion

In this study, by comparing phylogenies of genomes and genomic elements, we show that the *inv* gene, as the location of the important Tet-island in *C. sui*s, appears to transport with the Tet-island during recombination events, rather than acting as an integration site. Additionally, the distribution of the Tet-island across the phylogeny does not lend itself to the inference of a simple vertical inheritance, and recombination must be invoked to explain the pattern most parsimoniously. Some recombination events appear to have occurred been between Clade 1 and Clade 2 (interclade). A high proportion of sequenced *C. suis* field strains carry the Tet-island (Fig. 1), which may represent bias in the strains selected for sequencing. Sadly, historical isolates were not collected, and the history of the insertion of the Tet-island remains unknown. We add further evidence of the high rates of recombination in *C. suis*, occurring with high frequency between the two main clades, and readily transferring the Tet-island.

### Electronic supplementary material

Below is the link to the electronic supplementary material.

Supplementary Material 1

Supplementary Material 2

Supplementary Material 3

Supplementary Material 4

## Data Availability

Sequence data used in this study has been made available with the following bioprojects: PRJNA668469 (66 strains) and PRJEB17986 (29 strains), as well as PRJNA326179 (11 strains) and PRJNA221336 (Strain ID: MD56). Detailed information on accession data is listed in the supplementary information (Table S1).
