# Supplementary material for: Chlamydia suis undergoes interclade recombination promoting Tet-island exchange
Source: BMC Genomics. 2024 Jul 26;25:724. doi: 10.1186/s12864-024-10606-6 (PMC11282597; doi:10.1186/s12864-024-10606-6)
Supplement: Supplementary file 4 — Supplementary Material 4 [file 12864_2024_10606_MOESM4_ESM.docx]

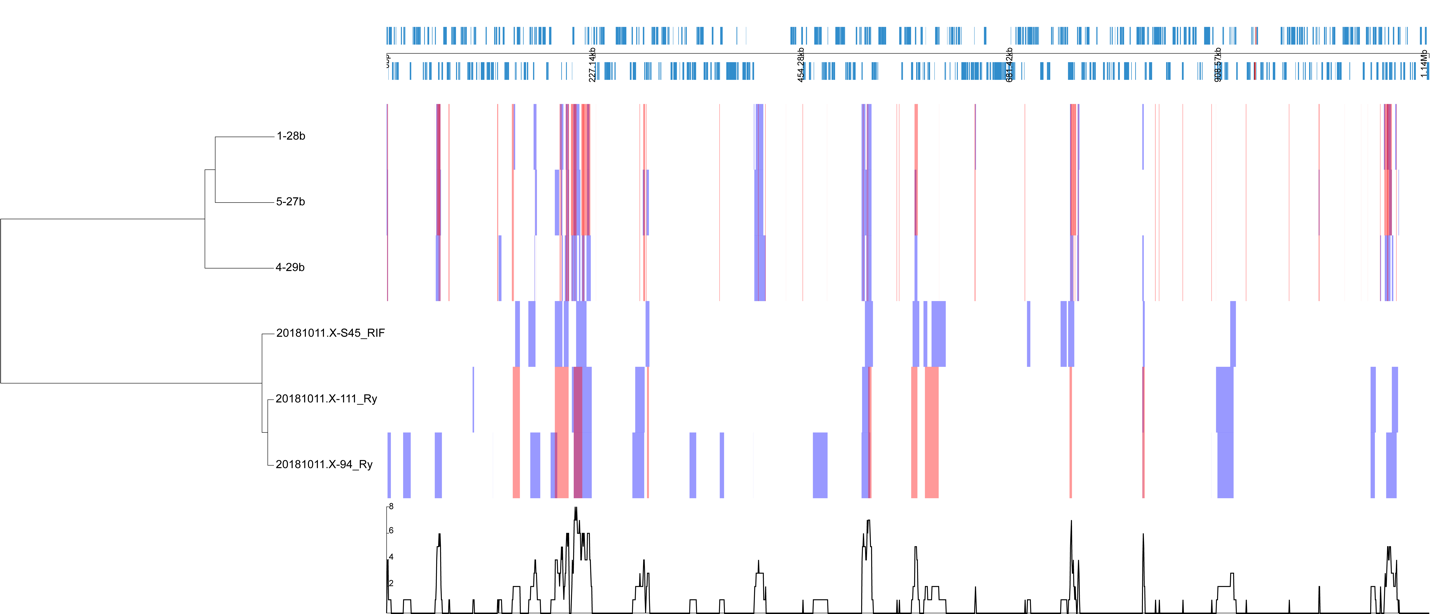


**Figure S1. Recombinations identified solely among parental strains from the in vitro experiment.** The sample names are matched to the genome length tracks (right) where red bars indicate recombinations identified in more than one genome, and blue bars show those only identified in single genomes. Below the tracks is a plot of recombination density.
